# Can’t keep it SECRET: system evaluation of carbapenem restriction against empirical therapy

**DOI:** 10.1093/jacamr/dlac137

**Published:** 2023-01-02

**Authors:** Drew A Wells, Asia J Johnson, Jack G Lukas, Diana A Hobbs, Kerry O Cleveland, Jennifer D Twilla, Athena L V Hobbs

**Affiliations:** Department of Pharmacy, Methodist Le Bonheur Healthcare—University Hospital, 1265 Union Avenue, Memphis, TN 38104, USA; Department of Clinical Pharmacy and Translational Science, University of Tennessee Health Science Center, 881 Madison Avenue, Memphis, TN, USA; Department of Pharmacy, Methodist Le Bonheur Healthcare—University Hospital, 1265 Union Avenue, Memphis, TN 38104, USA; Department of Pharmacy, Methodist Le Bonheur Healthcare—University Hospital, 1265 Union Avenue, Memphis, TN 38104, USA; Department of Radiology, Washington University School of Medicine, 4525 Scott Avenue, St Louis, MO 63110, USA; Infectious Diseases, Methodist Le Bonheur Healthcare—University Hospital, 1265 Union Avenue, Memphis, TN 38104, USA; Division of Infectious Diseases, University of Tennessee Health Science Center, 1325 Eastmoreland Avenue, Suite 460, Memphis, TN 38104, USA; Department of Pharmacy, Methodist Le Bonheur Healthcare—University Hospital, 1265 Union Avenue, Memphis, TN 38104, USA; Department of Clinical Pharmacy and Translational Science, University of Tennessee Health Science Center, 881 Madison Avenue, Memphis, TN, USA; Cardinal Health, 13651 Dublin Court, Stafford, TX 77477, USA

## Abstract

**Objectives:**

Carbapenems are appealing agents for empirical use given their broad spectrum of activity; however, selective use is vital in minimizing the risk for development of carbapenem-resistant pathogens. We aimed to examine the impact of carbapenem restriction criteria and a pre-authorization process on utilization and cost savings across a health system.

**Methods:**

This retrospective study was conducted across five adult hospitals. The pre-implementation period was 8 February 2020 to 30 April 2020 and the post-implementation period was 8 February 2022 to 30 April 2022. The primary outcome was to compare the number of orders for carbapenems between the study periods for both the intervention and non-intervention hospitals. Secondary outcomes included projected annual cost and an estimated cost-savings evaluation using a stratified analysis for the intervention and non-intervention facilities to account for more resource-limited settings.

**Results:**

The total number of carbapenem orders decreased between study periods at the intervention hospital (246 versus 61, *P* < 0.01). At the non-intervention hospitals, orders decreased, although not significantly (333 versus 279, *P* = 0.58). Meropenem orders decreased by 66% compared with 12% for the intervention and the non-intervention hospitals, respectively (*P* < 0.001). Annual estimated cost for all facilities was $255 561 in the pre-implementation period compared with $29 593 in the post-implementation period (*P* < 0.001). Using a stratified analysis to account for available resources, the estimated annual cost saving was $225 968 for the system.

**Conclusions:**

Implementation of carbapenem restriction at the intervention hospital decreased utilization and provided significant cost savings. Furthermore, resource-limited facilities can still experience significant cost savings using a stratified antimicrobial stewardship intervention approach.

## Introduction

Pre-authorization and formulary restriction are two methods by which antimicrobial stewardship programmes (ASPs) can improve antimicrobial utilization.^1,2^ Use of broad-spectrum antibiotics, including carbapenems, contributes to antimicrobial resistance and development of MDR organisms (MDROs).^3^ Additionally, infections caused by MDROs are associated with longer hospital lengths of stay (LOS), increased *Clostridioides difficile* infections (CDI), and higher overall hospitals costs.^3–5^ Exposure to carbapenems, even for a short course, is a risk factor for the development of an infection caused by a carbapenem-resistant Gram-negative pathogen.^6,7^ Thus, antimicrobial stewardship efforts to reduce inappropriate carbapenem use are vital to mitigate the emerging risk posed by carbapenem-resistant pathogens, including carbapenem-resistant *Pseudomonas aeruginosa* (CRPA), Enterobacterales (CRE) and *Acinetobacter baumannii* (CRAB).

There is limited evidence directly comparing pre-authorization practices within a health system and the impact on the real-time utilization and cost. Improving utilization is surmised to have downstream effects on clinical outcomes such as development of carbapenem-resistant pathogens. Therefore, we sought to evaluate the impact of carbapenem restriction criteria on overall utilization and estimate the economic effect in a large health system.

## Materials and methods

### Study design and patient population

This multicentre, retrospective study was conducted across five adult hospitals in a single healthcare system. The pre-implementation period was 8 February 2020 to 30 April 2020 and the post-implementation period was 8 February 2022 to 30 April 2022. A computer-generated report was used to identify all patients who received a carbapenem during the pre-implementation and post-implementation periods. Patients were included if they were at least 18 years old and received at least 24 h of meropenem or ertapenem, which were the formulary carbapenems in the healthcare system. Patients were excluded if they received meropenem or ertapenem intraoperatively or for perioperative prophylaxis, were aged greater than 90 years, or were pregnant. Patient data were collected retrospectively in the Cerner Millennium^®^ electronic medical record (EMR) and managed using the REDCap^™^ (Research Electronic Data Capture) electronic data capture tools hosted at Methodist Le Bonheur Healthcare (MLH).^8,9^

### Restriction criteria implementation

A medication use evaluation (MUE) conducted within the system in the fall of 2021 demonstrated that 67% of patients received carbapenem therapy inappropriately, the majority of which were used empirically with no culture data to warrant a carbapenem as initial therapy.^10^ Carbapenem therapy during the initial MUE was deemed appropriate in patients with an active ESBL infection, history of an ESBL or antipseudomonal antibiotic-resistant organism within the previous 12 months of admission, or clinically worsening (based on provider discretion) despite receiving 48 h of either cefepime or piperacillin/tazobactam. The results of this MUE prompted the system ASP to develop carbapenem restriction criteria (Table 1).

**Table 1.** Restriction criteria for carbapenem use at the intervention hospital

Meropenem	Ertapenem (*ONE time dose ONLY*)
Active ESBL infection History of ESBL within 3 months Clinically worsening after 48 h of either piperacillin/tazobactam or cefepime Septic shock (i.e. sepsis & lactate >2 mmol/L & vasopressor use) and high suspicion of ESBL infection approved 48 h pending culture results Intra-abdominal infection with severe anaphylactic penicillin allergy	Pre-operative antibiotic prophylaxis Patients requiring outpatient ertapenem therapy may receive one dose on the day of discharge

ESBL, extended-spectrum beta-lactamase-producing organism.

These restriction criteria were implemented through a pre-authorization process on 8 February 2022 at the health system’s flagship hospital, an academic medical centre with 583 licensed beds, which served as the intervention hospital for our study. The other four adult hospitals in the system were non-academic and were the comparator non-intervention hospitals in this study, totalling 828 licensed beds. None of the adult hospitals in the system met criteria to be a critical access hospital, and all of the non-intervention facilities had on-site infectious diseases (ID) physicians available. The pre-authorization process was a two-step process consisting of ordering providers being prompted to complete an electronic order form and select the applicable criteria for use (Table 1) for the patient. Subsequently, the pharmacist reviewing the order would validate the criteria for use were appropriate and document using the pharmacist verification form prior to verifying the carbapenem order. If the pharmacist review was inconsistent with the restriction form the provider completed, the pharmacist would contact the provider and recommend an appropriate alternative antimicrobial agent based on an algorithm that was approved by the system ASP. In instances when the provider insisted on using a carbapenem for a patient who did not meet the pre-specified criteria for use, the pharmacist verified the order until noon the next business day and placed a consult for the clinical pharmacist to review and follow-up with the provider. ID physicians were also required to complete the physician order form. All orders for carbapenems entered by ID physicians at the intervention facility were verified upon order entry and retrospectively reviewed and addressed if necessary by the ID clinical pharmacy specialist within the next business day. Finally, an ID physician and ID-trained pharmacist were available at the intervention facility for consultation and intervention in cases where there was still disagreement between non-ID providers and the clinical pharmacist. All documentation on the provider order form and pharmacist verification form was available in the EMR.

Lastly, provider and pharmacist education regarding the restriction criteria and pre-authorization process was created and conducted at the intervention hospital in January 2022 by the ID clinical pharmacy specialist, the PGY2 Internal Medicine pharmacy resident, and a PGY1 pharmacy resident. Education included presentations, meetings and emails to key stakeholders focusing on the internal data and literature supporting the carbapenem criteria for use. Targeted provider groups included ID, solid organ transplant, bone marrow transplant, emergency medicine, critical care and internal medicine. All pharmacists were required to complete a competency-based electronic training module focused on the utilization criteria and pre-authorization process.

### Outcomes

The primary outcome was to compare the number of active orders for carbapenems between the pre-implementation and post-implementation periods for both the intervention and non-intervention hospitals. Additional secondary outcomes included evaluation of estimated annual cost between study periods as well as an estimated cost-savings evaluation across the entire system using a stratified analysis for the intervention and non-intervention facilities. Carbapenem cost in the pre- and post-implementation periods was calculated by accounting for the total number of carbapenem orders multiplied by median duration of therapy observed at the intervention hospital (5.8 days pre-implementation and 2.4 days post-implementation, *P* < 0.001).^10^ Estimated annual cost was determined by multiplying the estimated cost in each 12 week study period by 4.33 to project a 52 week cost savings. Of note, this cost-savings estimate did not include the cost gap of utilizing other antibiotics. During the study time frame, total daily cost of meropenem and ertapenem was $5.76 and $36.03, respectively. For the intervention hospital, the estimated annual cost saving was calculated based on the reduction in utilization with the implementation of the restriction criteria for both meropenem and ertapenem. For the non-intervention hospitals with fewer clinical pharmacy resources, a stratified analysis was performed to project cost savings if only an inpatient conversion from ertapenem to meropenem was used. Cost savings for this analysis were conducted by first calculating the percent reduction of ertapenem orders at the intervention hospital then converting the same percent orders from ertapenem to meropenem at the non-intervention hospitals.

### Statistical analysis

Baseline characteristics and number of carbapenem orders were analysed using chi-squared and *t*-tests. Total cost differences were compared between both study periods using Student’s *t*-tests and Mann–Whitney *U*-tests. Results were considered statistically significant with *P* values less than 0.05. Data were analysed using the statistical software package R version 4.0.3 (R Development Core Team, 2020).

### Ethics

The University of Tennessee Health Science Center Institutional Review Board approved this study for exempt review (22-08708-XM). No informed patient consent was required for purposes of this study due to its retrospective nature.

## Results

**Figure 1.**
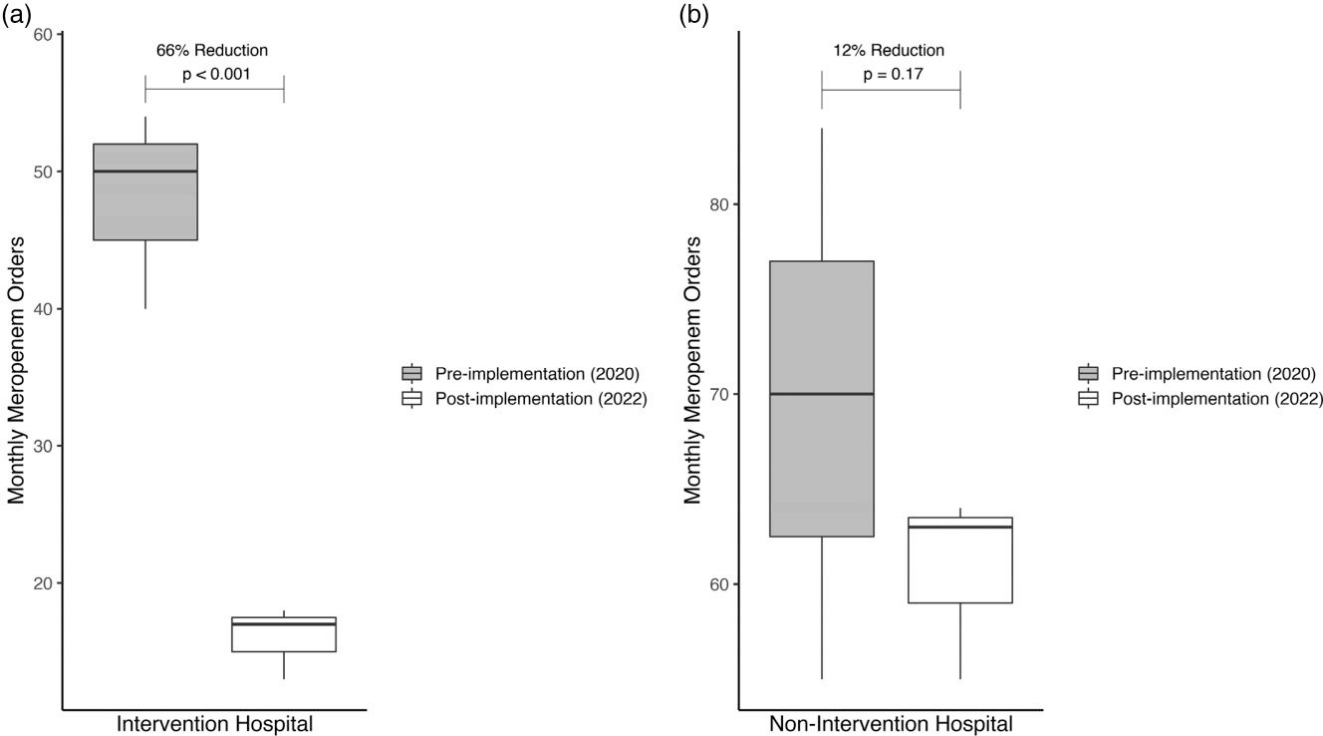
Percent reduction of monthly meropenem orders between the intervention (a) and non-intervention (b) hospitals.

Across all five adult hospitals, there were a total of 579 active carbapenem orders in the pre-implementation period and 340 orders in the post-implementation period. Baseline characteristics were similar between both periods (Table 2). For the primary outcome, the total number of carbapenem orders at the intervention hospital decreased from 246 in the pre-implementation period to 61 in the post-implementation period (*P* < 0.01). Total number of orders decreased from 333 to 279 between study periods at the non-intervention hospitals, but this was not significant (*P* = 0.58, Table 3). Meropenem orders were decreased by 66% compared with 12% at the intervention and non-intervention hospitals, respectively (*P* < 0.001, Figure 1).

**Table 2.** Baseline characteristics

Patient characteristics	Pre-implementation period (*n* = 579)	Post-implementation period (*n* = 340)	*P* value
Age, years, median (IQR)	62 (52–73)	62 (48–73)	0.20
Female	315 (54.4)	177 (52.1)	0.54
Antibiotic allergies			
Penicillin	63 (10.9)	78 (22.9)	0.21
Cephalosporin	9 (1.6)	7 (2.1)	0.62
Other^a^	37 (6.4)	36 (10.6)	0.91

All data represented as *n* (%) unless otherwise stated.

a Other includes aminoglycosides, clindamycin, daptomycin, fluoroquinolones, linezolid, sulphonamides, tetracyclines and vancomycin.

**Table 3.** Primary and secondary outcomes compared between pre-implementation and post-implementations periods

	Pre-implementation period (*n* = 579)	Post-implementation period (*n* = 340)	*P* value
Primary outcome			
Total number of carbapenem orders	579	340	<0.001
Number of orders at the intervention hospital	246	61	<0.01
Meropenem	144 (58.5)	48 (78.7)	<0.001
Ertapenem	102 (41.5)	13 (21.3)	<0.001
Number of orders at the non-intervention hospitals	333	279	0.58
Meropenem	209 (62.8)	182 (65.2)	0.17
Ertapenem	124 (37.2)	97 (34.8)	0.07
Secondary outcomes^a^			
Total estimated annual cost for the system, $	255 562	29 594	<0.001
Total estimated annual cost at the intervention hospital, $	113 126	7860	<0.001
Total estimated annual cost at the non-intervention hospitals^a^, $	142 436	21 733	<0.001

All data represented as *n* (%) unless otherwise stated.

a Estimated cost assumes non-intervention hospital converted the majority of ertapenem orders to meropenem assuming the same total number of orders.

**Figure 2.**
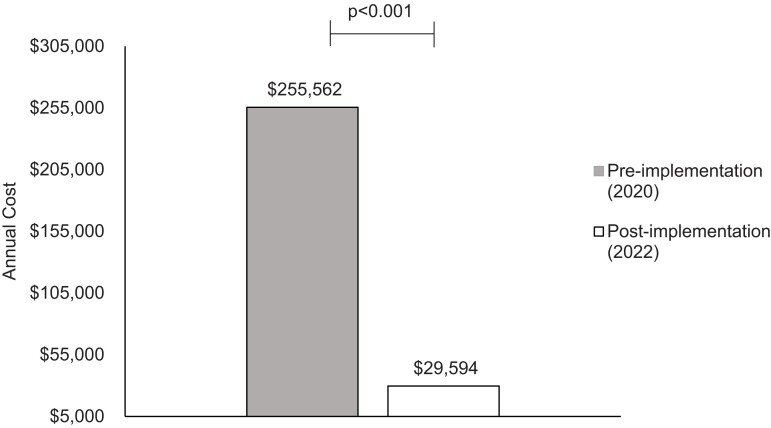
Projected annual cost based on the cost savings between the pre-implementation and post-implementation periods.

For the secondary outcomes, annual estimated cost of carbapenem therapy for all facilities was $255 562 in the pre-implementation period compared with $29 594 in the post-implementation period (*P* < 0.001), based on the stratified cost-savings analysis (Figure 2). The projected annual cost saving was $105 266 at the intervention hospital and $120 702 at the non-intervention hospitals, for a total of $225 968 within the system (Table 3).

## Discussion

Our study results highlight the positive impact that a pre-authorization process and restriction criteria can have to decrease inappropriate carbapenem utilization and provide cost savings. According to the CDC, CRE and CRAB are urgent public health threats that require aggressive measures to combat.^3^ These pathogens are difficult to treat with available antibiotics and often require treatment with newer therapies including ceftolozane/tazobactam, meropenem/vaborbactam, ceftazidime/avibactam, imipenem/cilastatin/relebactam and cefiderocol. With daily acquisition costs ranging from $668 to $1076 per day, these agents are more expensive than carbapenems, which can restrict their availability in resource-limited settings.^11^ The prohibitive cost of these agents and their limited access further emphasize the pressing need to minimize empirical use of carbapenems to help mitigate the development of carbapenem-resistant pathogens.

Previous studies have demonstrated that implementation of carbapenem restriction criteria results in an overall reduction of carbapenem utilization and enhanced carbapenem susceptibilities. Bantar *et al*.^12^ showed that restriction criteria decreased carbapenem use from 13.5 to 6.2 DDD per 1000 patient days (DDD/1000 PD, *P* = 0.03) and decreased the proportion of imipenem-resistant *P. aeruginosa* isolates (19% versus 0%, *P* = 0.02). White *et al*.^13^ reported similar findings, in that decreasing imipenem use through restrictions led to improvement in *P. aeruginosa* susceptibilities for the inpatient setting (83% to 95%, *P* ≤ 0.01) and the ICUs (65% to 83%, *P* ≤ 0.01). Pakyz *et al*.^14^ evaluated the impact of carbapenem restriction on utilization and proportion of CRPA in academic medical centres and demonstrated that overall carbapenem use decreased significantly (*P* = 0.04) and reported a lower proportion of CRPA (*P* = 0.01) across the 5 year study. The limited time frame in our study did not allow for assessment of carbapenem susceptibilities. However, unlike other studies, we were able to control for time bias by evaluating the effect of carbapenem restriction criteria and pre-authorization on utilization between the intervention and non-intervention hospitals within the same time frame. Furthermore, the initial confirmation of the provider-completed restriction form was conducted by the pharmacist at order verification, thus engaging the entire pharmacy department in this initiative instead of relying solely on an ID-trained pharmacist or ID physician.

The decrease in the number of carbapenem orders at the intervention hospital is likely to be multifactorial. The pre-authorization process requiring the ordering provider to complete the restriction form may deter inappropriate use due to lack of an indication by increasing the number of steps required for providers to order a carbapenem. Additionally, the creation of the restriction criteria and education of key stakeholders by the system ASP, co-led by an ID physician and pharmacist, may have increased provider buy-in and compliance. Unfortunately, restriction forms can be circumvented by providers listing an unconfirmed or differential diagnosis to meet the requirements for use. In our process, this was mitigated by the pharmacist verifying the order, who was required to complete their own documentation validating the criteria-for-use form completed by the provider. For instances where patients did not meet criteria despite the form suggesting the patient does, the pharmacist would contact the provider and recommend an appropriate alternative therapy. By building in safety measures that allowed providers to override the carbapenem restriction through a discussion with the order verification pharmacist in the event that the patient did not meet criteria, our restriction programme empowered every pharmacist in the department to intervene at the point of order verification. This strategy prevented many patients from inappropriately receiving empirical carbapenem therapy that would otherwise have been initiated during the evening or weekend hours when clinical pharmacy staff were not available to discuss the use of alternative agents with providers. An additional benefit of this tactic prevented carbapenems from being continued in patients without a clear indication due to the clinical inertia argument that the patient was stable on the initial antibiotic therapy.

Additionally, cost savings associated with carbapenem restriction is an attractive benefit to both large healthcare systems and smaller or resource-limited facilities. The restriction criteria at the intervention hospital were associated with over $100 000 in estimated annual savings in our study. For large healthcare systems that have ASPs led by ID physicians and pharmacists, and/or adequate staff to institute such restriction criteria, significant cost savings are attainable by implementing a similar process. However, we recognize that smaller facilities such as some of the critical access hospitals in our own healthcare system may not have pharmacist staffing capacity on site to confirm criteria for use prior to order verification, and that implementation of such a robust restriction programme is not always feasible. In these instances, inpatient formulary restriction of ertapenem through conversion to meropenem can also provide significant cost savings, though impact on development of carbapenem resistance will likely be negligible. At the non-intervention hospitals, selective inpatient use of meropenem over ertapenem would have resulted in approximately $120 000 in annual savings, even without the implementation of the restrictive carbapenem criteria for use. This formulary restriction strategy provides smaller institutions an opportunity to significantly reduce carbapenem cost and streamline the inpatient formulary. Extrapolation of the stratified restriction criteria presented an annual savings opportunity of over $225 000 within our healthcare system.

Notable limitations to our study are largely related to the retrospective nature of our review. Our analysis is limited to practical outcomes, including decreasing utilization and cost savings, with no assessment of clinical outcomes including adverse effects, selected alternative therapy, clinical response, or antimicrobial resistance. Additionally, the calculation of cost in the pre- and post-implementation periods is limited since the duration of therapy was extrapolated from the previous study at the intervention hospital. In terms of cost savings, the cost of alternative therapy could not be calculated since alternative therapy was not evaluated in this study. Furthermore, our inability to evaluate carbapenem resistance trends in response to the restriction programme precluded us from calculating additional cost-saving opportunities such as avoidance of newer and more expensive therapies, longer hospital LOS, and higher mortality expected in patients with infections due to carbapenem-resistant pathogens.

### Conclusions

Implementation of carbapenem restriction criteria at the intervention hospital decreased carbapenem utilization and provided significant cost savings compared with the non-intervention hospitals.
